# Isomaltooligosaccharides inhibit early colorectal carcinogenesis in a 1,2-dimethylhydrazine-induced rat model

**DOI:** 10.3389/fnut.2022.995126

**Published:** 2022-09-15

**Authors:** Xiao Chen, Shaoli Li, Cuixia Lin, Zhen Zhang, Xiaoyan Liu, Chunhui Wang, Jun Chen, Binbin Yang, Jing Yuan, Zheng Zhang

**Affiliations:** ^1^College of Health Sciences, Shandong University of Traditional Chinese Medicine, Jinan, China; ^2^Capital Institute of Pediatrics, Beijing, China; ^3^Chinese Medicine Innovation Research Institute, Shandong University of Traditional Chinese Medicine, Jinan, China; ^4^Department of Laboratory and Pathology, Armed Police Beijing Corps Hospital, Beijing, China; ^5^State Key Laboratory of Biobased Material and Green Papermaking, School of Food Science and Engineering, Qilu University of Technology, Shandong Academy of Sciences, Jinan, China

**Keywords:** isomaltooligosaccharides, colorectal cancer, gut microbiome, fecal metabolome, pathological analysis

## Abstract

Colon cancer (CC) is a multistage disease and one of the most common cancers worldwide. Establishing an effective treatment strategies of early colon cancer is of great significance for preventing its development and reducing mortality. The occurrence of colon cancer is closely related to changes in the intestinal flora structure. Therefore, remodelling the intestinal flora structure through prebiotics is a powerful approach for preventing and treating the occurrence and development of colon cancer. Isomaltooligosaccharides (IMOs) are often found in fermented foods and can directly reach the gut for use by microorganisms. In this study, a rat model of early colon cancer (DMH) was established by subcutaneous injection of 1,2-dimethylhydrazine, and the model rats were fed IMOs as a dietary intervention (DI). The untargeted faecal metabolomics, gut metabolome and intestinal function of the model rats were investigated. The results showed that DMH, DI and IMOs alone (IMOs) groups exhibited gut microbial community changes. In the DI group, there was an increased abundance of probiotics (*Lactobacillus*) and decreased abundance of CC marker bacteria (*Fusobacterium*). The key variations in the faecal metabolites of the DI group included decreased levels of glucose, bile acids (including deoxycholic acid and chenodeoxycholic acid) and amino acids (including L-glutamic acid and L-alanine). In addition, dietary intake of IMOs attenuated the intestinal inflammatory response, improved the intestinal microecological environment, and slowed the development of DMH-induced early CC in rats. This work provides a theoretical basis and technical support for the clinical prevention or treatment of CC with prebiotics.

## Introduction

Cancer is a global issue. Colorectal cancer (CRC) is the second most common cancer in women and the third most common cancer in men (1). The microbes in the gut of healthy adults are large and complex, numbering as many as 10^12^–10^14^ (2). The intestinal microbial structure is altered in CRC and early-stage colon cancer (ECC) (3). Compared to the healthy population, patients with CRC have pathological imbalances in the gut microbiota (4). In addition, there is a corresponding change in the metabolome during the progression of CRC (5). Intestinal microorganisms and their metabolites are closely connected with the occurrence and development of CRC. Therefore, regulating changes in the gut microbiota structure and monitoring the metabolites associated with CRC may cause the discovery of new ideas to prevent CRC.

A number of studies have reported that functional oligosaccharides can prevent and alleviate disease through targeted regulation of the gut microbiota (6, 7). Isomaltooligosaccharides (IMOs) are low-degree polysaccharides with at least one α-(1 → 6) glycosidic bond between glucose residues and a monosaccharide number of 2–5; IMO is partially digested *in vivo* by brush border maltase/glucoamylase and isomaltase (8–10). Undigested oligosaccharides are fermented in the large intestine, resulting in beneficial gastrointestinal effects and prebiotic properties (11). IMOs naturally occur in some fermented foods. Research indicates that IMOs can significantly inhibit the expression of IL-1 β in the cecum and colon of rats, increase the relative abundance of beneficial bacteria (such as *Bifidobacterium spp.* and *Lactobacillus spp.*), increase the content of Short chain fatty acids (SCFAs) in the cecum, and reduce experimental colitis in rats (12). In addition, there is evidence that IMOs can alleviate intestinal diseases for example constipation and irritable bowel syndrome (13–15).

The high mortality rate of colon cancer (CC) worldwide is related to the difficulty of identifying ECC. Most CC patients are in a late disease stage when they are diagnosed, which seriously impacts the survival rate and treatment effect. The aim of this work was to evaluate the intervention effects of IMOs supplementation against early CRC lesions in a 1,2-dimethyl hydrazine dihydrochloride (DMH)-initiated rat model and to investigate the effects on the biomarkers, intestinal microbiome and faecal metabolomics of CRC.

## Materials and methods

### Animals and diets

Male Wistar rats (6 weeks old, *n* = 32) were obtained from the Laboratory Animal Centre, Academy of Military Medical Sciences (Beijing, China) and were housed individually in a temperature- and humidity-controlled room (21 ± 3°C and 55 ± 5%, respectively) under a strict 12 h light/dark cycle. The rats were acclimated to their new surroundings for 1 week and were fed a standard chow diet (AIN-93M, American Institute of Nutrition) and provided water *ad libitum*. The protocols of experimental adopted in this work were approved by the Ethics Committee of the Shandong University of Traditional Chinese Medicine. The procedures adhered to the European Community guidelines for the care and use of laboratory animals.

IMOs were kindly obtain from Baolingbao Biology Co., Ltd. (Shangdong, China); the content of effective components (isomaltose, isomaltotriose and panose) was greater than 95%. All experimental diets were irradiated with γ-rays (25 kGy) and were based on the AIN-93M purified diet standard. IMOs were supplemented in the intervention diet by partially replacing the cornstarch component of the AIN-93M diet in equal amounts. Rat chows were provided by BiotechHD Co., Ltd. (Beijing, China).

### Experimental protocols

**FIGURE 1:**
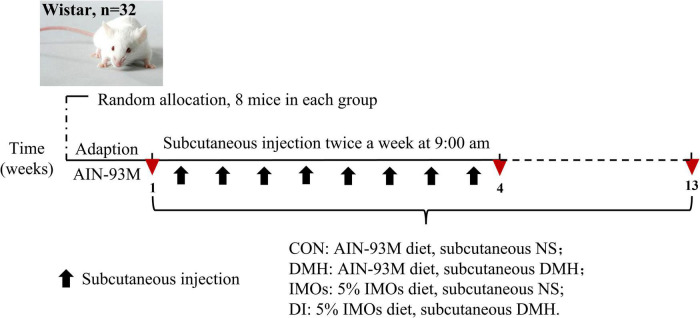
Experimental design.

After the acclimation period, 32 rats were randomly divided into four groups as follows: blank control (standard chow, CON), model (standard chow, DMH), intervention (diet supplemented with 5% IMOs, DI), control (diet supplemented with 5% IMOs, IMOs). The DMH and DI groups performed subcutaneous injections of DMH (Sigma-Aldrich, St Louis, MO, USA). The DMH was dissolved in normal saline and pH-adjusted to 6.5 using 0.25 M NaOH; rats received 40 mg/kg body weight injections twice a week for 4 weeks (16). Meanwhile, the CON and IMOs groups received similar subcutaneous injections of normal saline (pH 6.5). IMOs supplementation continued during DMH administration (Figure 1). The body weight and food intake changes of the rats were recorded weekly throughout the experiment.

### Sample collection

After 12 weeks of experiment, the rats were placed in individual sterilised metabolic cages. Their faecal pellets were collected in separate sterilised microcentrifuge tubes placed on ice, then immediately frozen in liquid nitrogen and stored at –80°C until analysis. The animals were killed after 12 h of fasting. Blood samples were obtained from the abdominal aorta, and serum was collected after centrifugation of blood at 1,000 *g* for 20 min at room temperature (24 ± 2°C). Colon tissue was removed via a longitudinal incision from the cecum to the anus after the rats were sacrificed. A random 1 cm sample taken from the middle of each rat colon was fixed in formalin for treatment, processed, and embedded in paraffin for histological observation. All remaining colonic tissue was sandwiched between filter papers and fixed in formalin overnight for aberrant crypt foci (ACF) analysis (17).

### Assessment of serum biochemical values

Serum tumour necrosis factor-α (TNF-α), interleukin-1β (IL-1β), interleukin-6 (IL-6), interleukin-8 (IL-8), d-lactate (D-LA) and diamine oxidase (DAO) levels were determined by enzyme-linked immunosorbent assay (ELISA) kits (Beijing Gersion Bio-Technology Co., Ltd., Beijing, China). Serum levels of glucose (GLC), total cholesterol (TC), albumin (ALB), low-density lipoprotein cholesterol (LDL-C), high-density lipoprotein cholesterol (HDL-C), triglycerides (TG), total protein (TP) and globulin (GLO) were analysed automatically with an auto-analyser (Au680, Beckman Coulter, USA) using commercial kits (Biosino Bio-technology and Science, Inc., Beijing, China).

### Aberrant crypt foci counting and faecal bacterial enzyme assays

The fixed colon tissue was stained with methylene blue (0.2%) in saline for 2–3 min. Samples were morphologically examined under an optical microscope (Olympus, Optical Co. Ltd., Tokyo, Japan) at 40 × magnification, and ACF were counted.

Faecal pre-treatment: fresh faecal samples were fully suspended in 0.1 M potassium phosphate buffer (pH 7.0), pre-cooled by a homogenizer, and then homogenized. The supernatant was obtained by ultrasound at 4°C for 10 s (six times in total) and centrifugation at 2,000 *g* for 15 min (18).

β-glucuronidase assay: (18, 19) β-glucuronidase activity in rat faeces was determined according to the consumption of 4-nitrophenyl-β-D-glucuronide (Sigma-Aldrich, St Louis, MO, USA) in the reaction system. The 1 ml reaction system contained 0.02 M potassium phosphate buffer (pH 7.0), 0.1 mm EDTA, 2 mm 4-nitrophenyl-β-d-glucosidic acid solution and 100 μl faecal supernatant; incubation was performed for 30 min at 37°C. Then, 0.2 M NaOH solution was added to stop the reaction, and the release of 4-nitrophenol was measured at 405 nm.

β-glucosidase assay: (18, 19) the procedure and reaction system for the β-glucosidase assay were the same as described above for the β-glucuronidase assay. The reaction substrate was 4-nitrophenyl-β-D-glucopyranoside (Bailingwei Technology Co., Ltd., Beijing, China).

### Colon contents pH assay

100 mg of colonic content was accurately weighed, suspended in 1.5 ml deionized water, and centrifuged at 18,000 *g* for 15 min. Then, a pH metre was used to measure the pH value of the supernatant (20).

### Histopathological examination of colonic tissues

Colonic tissues were dissected and fixed in 10% phosphate-buffered formaldehyde solution (Beijing Solarbio Life Sciences Co., Ltd., Beijing, China), then embedded in paraffin and cut into sections for Periodic Acid-Schiff (PAS), haematoxylin and eosin (H&E) and immunohistochemical (IHC) staining. Monoclonal antibodies for inducible nitric oxide synthase (iNOS), Cyclooxygenase-2 (COX-2) and beta-Catenin (β-Catenin; 1:200, OmnimAbs Biotech Co., Ltd., Alhambra, CA, USA) were used to evaluate the expressions of COX-2, iNOS and β-Catenin in IHC-paraffin sections (IHC-P) of colonic mucosal epithelium from the rats. Microscopic data for the positive control cells (tan-yellow cytoplasm) of COX-2, iNOS and β-Catenin in the colon were analysed and measured by Image Pro-Plus 6.0 software (Media Cybernetics, lnc., Rockville, MD, USA). The protein expression levels were measured in according to the method described by Mao et al. (21).

### Faecal microbiota analysis by 16S rRNA gene sequencing

Faecal microbiome DNA was extracted in accordance with the DNeasy Powersoil kit instructions (Qiagen, GmbH, Hilden, Germany). The longitudinal sample processing, Illumina MiSeq sequencing, and library generation procedures were as described previously (6). The bacterial diversity analysis in this study was based on amplification of the V3-V4 hypervariable region of the 16S ribosomal RNA (rRNA) gene. The primer sequences were as follows: 343F: 5′-TACGGRAGGCAGCAG-3′, 798R: 5′-AGGGTATCTAATCCT-3′. High-fidelity DNA polymerase (ExTaq, Takara, Japan) was used for PCR amplification to ensure efficient and accurate amplification. After purification, the PCR products were amplified twice, and then paired-end (PE) sequencing was performed.

Raw sequencing data was preprocessed in FASTQ format by Trimmomatic software (22) to filter low quality reads. As previously described, (6) processed sequence reads were assembled using FLASH software (23). The Quantitative Insights Into Microbial Ecology (QIIME, version 1.8.0) wrapper was used to assign 75% of sequence reads with a base quality score > 20 (99% base calling accuracy) to classification (24). Used the workflow provided by the QIIME wrapper to select operational taxonomic units (OTUs) from valid labels with a similarity threshold of 97% (25). identified and annotated all representative reads with a confidence threshold of 70% by comparing the basic local alignment search tool with the Greengenes (16S rRNA) database using the Ribosomal Database Project II classifier (26).

### Faecal metabolite analysis

Faecal proteins were separated with methanol and chloroform, and 20 μl of L-2-chlorophenylalanine solution (1 mg/ml in dd water) was added as an internal standard. After homogenization and sonication, the supernatant was transferred to gas chromatography/mass spectrometry (GC/MS) glass vials for coupled identification of gut microbial hosts using an Agilent 7890A GC system (Agilent Technologies, Santa Clara, CA, USA). Related metabolites were analyzed according to previously reported methods using Pegasus HT time-of-flight (TOF)-MS (Leco, Saint Joseph, MI, USA) (27).

### Statistical analysis

All samples were tested in triplicate unless otherwise indicated. Data are shown as mean ± standard error of the mean (mean ± s.e.m.) in line and bar graphs. Box plots represent median values with a midline, and the lower and upper edges of the box reflect the 25th and 75th quartiles (25| 75), respectively. For comparison of independent measurements, *t*-tests were performed as appropriate using Statistical Products and Services Solutions (SPSS Inc., Chicago, IL, USA; version 22.0). Significance was defined as *p* < 0.05. Multivariate analyses (principal component analysis [PCA]) were performed using the SIMCA 14.1 software wrappers (MKS Data Analytics Solutions, Umea, Sweden).

## Results

### Body weight, food intake, serum biochemical values and inflammatory factors

The average daily food and energy intake of rats in the four groups are shown in Supplementary Table 1, and the rats’ weekly body weight changes during the experiment were shown in Supplementary Figure 1. Average daily food and energy intake did not differ significantly between the four groups (*p* > 0.05). However, after 2 weeks of DMH treatment, the body weights of rats in the DMH and DI groups were significantly lower than that of the CON group (*p* < 0.05). In the fourth week, the average body weight of DMH-treated rats that also received the IMOs diet intervention had gradually recovered (DI group). In the sixth week, the DI group rats had a significantly higher average body weight than the DMH group (*p* < 0.05). At the end of the experiment, there was no significant difference in body weight between the CON and IMOs groups. The average body weight of the DMH group was significantly lower than the other three groups (*p* < 0.05). The average body weight of the DI group was significantly higher than that of the DMH group (*p* < 0.05), but lower than that of the CON and IMOs groups.

Serum biochemical indicators were determined in the CON, DMH, IMOs and DI groups after 12 weeks of treatment (Table 1). LDL-C and GLO levels in the DI-treated rat serum were significantly lower than those of the DMH-treated rats (*p* < 0.05). Other serum parameters, including TP, ALB, TG, TC, HDL-C and GLU, did not significantly differ between the DMH and DI groups (*p* > 0.05). The IMO-treated rats tended to have lower blood glucose levels than the control group. Previously, it was generally believed that IMOs is a prebiotic that can regulate the intestinal microbiota. However, a new study pointed out that IMOs is a slowly digested carbohydrate and therefore can regulate blood glucose (28).

**TABLE 1:** Clinical chemistry data from serum samples after 12 weeks of treatment.

	CON	DMH	IMOs	DI
TP (mmol/L)	63.77 ± 4.78^ab^	66.67 ± 3.05^b^	62.51 ± 1.94^a^	65.98 ± 1.81^b^
ALB (mmol/L)	36.13 ± 3.14	35.68 ± 2.27	36.45 ± 1.07	36.56 ± 1.22
TG (mmol/L)	1.71 ± 0.49^b^	0.93 ± 0.56^a^	1.50 ± 0.37^ab^	1.34 ± 0.69^ab^
CHO (mmol/L)	2.02 ± 0.76^b^	1.35 ± 0.34^a^	1.48 ± 0.41^a^	1.39 ± 0.17^a^
HDL-C (mmol/L)	1.64 ± 0.59^b^	1.15 ± 0.17^a^	1.59 ± 0.32^b^	1.18 ± 0.29^a^
LDL-C (mmol/L)	0.36 ± 0.18^b^	0.24 ± 0.05^b^	0.19 ± 0.05^a^	0.19 ± 0.04^a^
GLU (mmol/L)	15.34 ± 2.89^b^	12.96 ± 3.53^ab^	11.85 ± 2.95^a^	10.79 ± 3.23^a^
GLO (mmol/L)	27.64 ± 1.71^a^	30.99 ± 2.35^b^	28.06 ± 1.81^a^	29.11 ± 2.03^a^

Values are presented as the mean ± s.e.m., *n* = 8; significant *p* values are presented horizontally. Significantly different values are denoted by letters.

**FIGURE 2:**
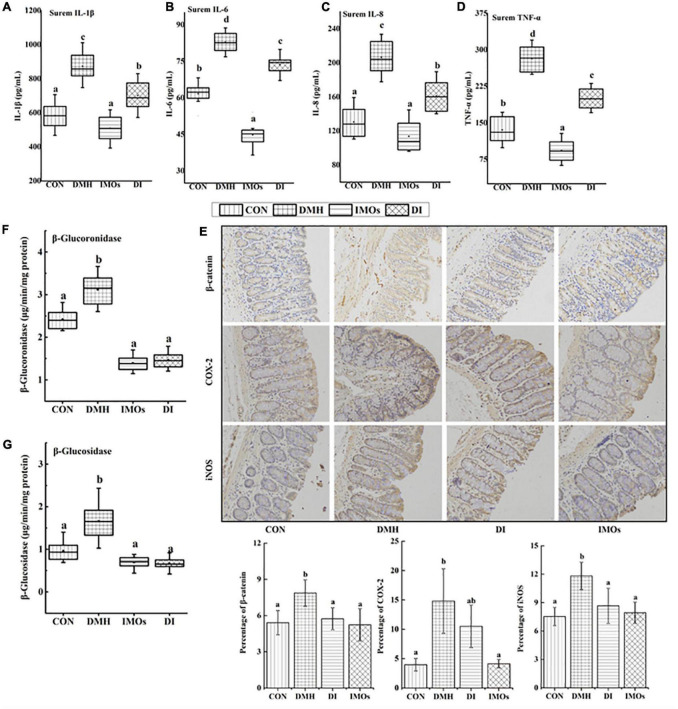
The levels of **(A)** IL-1 β, **(B)** IL-6, **(C)** IL-8 and **(D)** TNF-α in rat serum. **(E)** Immunohistochemistry of β-catenin, COX-2 and iNOS in rat colonic tissue. β-glucoronidase and positive expression rates of β-catenin, COX-2 and iNOS in colonic tissues were analysed using Image Pro-Plus 6.0 software. The levels of **(F)** β-glucoronidase and **(G)** β-glucosidase in rat faeces. Values followed by different letters (a, b and c) in each group are significantly different (*p* < 0.05).

Chronic intestinal inflammation is an important risk factor for CC. Chronic intestinal inflammation is closely related to the expression of pro-inflammatory factors such as TNF-α, IL-1β, IL-6 and IL-8. Cytokines can change the signal transduction pathway of tumour cells via paracrine signalling to regulate tumour growth. For example, TNF and IL-1 can activate NF-κB, and IL-6 activates signal transducer and activator of transcription 3 (29). In this study, the serum levels of TNF-α, IL-1β, IL-6 and IL-8 were detected in rats with ECC after 12 weeks of dietary intervention with IMOs, as shown in Figure 2. Compared with the CON group, the serum levels of TNF-α, IL-1β, IL-6 and IL-8 in the DMH group were significantly increased (*p* < 0.05). However, the serum levels of inflammatory factors in the DI group were significantly lower than those in the DMH group (*p* < 0.05). These results indicate that dietary supplementation with IMOs can significantly mitigate the increase in serum inflammatory factors in rats due to ECC.

### Individual effects of isomaltooligosaccharides on aberrant crypt foci and bacterial enzymes

**FIGURE 3:**
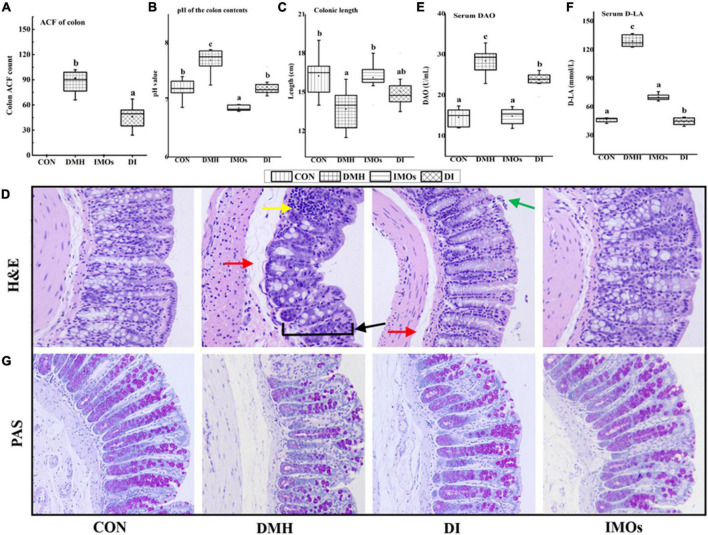
**(A)** Colon ACF counts among rats in the four groups. **(B)** Effect of IMOs treatment on pH of colonic contents in rats. **(C)** Effect of IMOs treatment on colon length in rats. **(D)** H&E staining of colonic tissue from rats (200× magnification). Effect of IMOs treatment on intestinal permeability in DMH-treated rats. The levels of **(E)** DAO and **(F)** D-LA in rat serum and **(G)** PAS staining of colonic tissue from rats (200× magnification). Values followed by different letters (a, b and c) in each group are significantly different (*p* < 0.05).

DMH-induced ACF in colonic mucosa is an obvious morphological change observed in the colons of rats. It is an important stage in the progression of CC and a pathological change associated with ECC. At the end of the 12-week experimental period, colonic ACF in the rats were counted and the results are shown in Figure 3A. ACF were detected in all DMH-treated groups (100% incidence), while no ACF were observed in the CON and IMOs groups. Dietary supplementation with IMOs caused significant inhibition of ACF formation (29.4%) with a significant reduction in the total number of ACF in the DI group (68.5 ± 19.5) as compared to the rats that did not receive IMOs supplementation (101.5 ± 35.5, *p* < 0.05; Figure 3A).

High levels of β-glucuronidase and β-glucosidase are generally considered to be biomarkers of CC. The levels of β-glucuronidase and β-glucosidase in the faeces of rats are shown in Figures 2F,G. Overall, there were higher enzyme activities in the faeces of rats in the DMH group. The DI group, which received IMOs supplementation, exhibited significantly (*p* < 0.05) lower concentrations of β-glucuronidase and β-glucosidase compared with DMH group. Similarly, the CON and IMOs groups also exhibited lower levels of β-glucuronidase and β-glucosidase compared with DMH group. No significant differences between CON, DI and IMOs groups (*p* > 0.05).

### Colonic function and morphology

The SCFAs acetate, propionate and butyrate are the main products of anaerobic fermentation of intestinal prebiotics; they cause the pH of the colon to decrease, and a decrease in the pH of the colon environment is beneficial to the growth of probiotics (30). This cycle contributes to a good intestinal microecological environment. In this study, the pH values of the colon contents of the four groups of rats were tested to investigate the effects of ECC and dietary supplementation of IMOs on the colon ecological environment of rats. The results are shown in Figure 3B. The pH value of the colon contents of the DMH group was significantly higher than that of the CON group (*p* < 0.05). The pH value of the colon contents of rats in the DI group was significantly lower than that of the DMH group (*p* < 0.05). Dietary supplementation with IMOs significantly reduced the increase in the pH value of the colon contents of rats with ECC. The pH value of the colon contents of rats in the IMOs group was significantly lower than that of the CON group (*p* < 0.05). Thus, dietary supplementation with IMOs can significantly reduce the pH value of the colon contents of healthy rats. This may be because IMOs, as prebiotics, promote the proliferation of certain probiotics (31), resulting in the production of more SCFAs in the intestines. This hypothesis remains to be tested in the future.

The colon length measurement results of rats in each experimental group are shown in Figure 3C. Compared with the CON group, the average colon length of rats in the DMH group was significantly shorter (*p* < 0.05), which is similar to the results of Kohno’s study (32). In addition, rats with inflammatory bowel disease exhibit colon shortening, but the pathological mechanism is not clear. In this study, dietary supplementation with IMOs tended to improve the colon shortening in rats with ECC, but the effect was not statistically significant. Compared with the CON group, the colon length of the IMOs group was not significantly different, and dietary supplementation with IMOs had no significant effect on the colon length of healthy rats.

The H&E staining results on the pathological sections of rat colon are shown in Figure 3D. The glands and thickness of the colonic mucosa were decreased in the DMH group (indicated by the black arrow). This was accompanied by diffuse infiltration of local inflammatory cells (indicated by the yellow arrow). The submucosa was oedematous, and the space was enlarged (indicated by the red arrow). In the DI group, the colonic submucosa was slightly oedematous (indicated by the red arrow), a small amount of mucosal epithelial cells was missing (indicated by the green arrow), and there was no other obvious damage. Dietary supplementation with IMOs had a certain repair effect on the colonic tissue damage in ECC model rats. The colon mucosa, submucosa, muscular layer and serosa layer of the CON group and IMOs group exhibited clear structures and boundaries. There were abundant mucosal glands, the tissue structure and intestinal wall thickness were intact, and there was no obvious damage. These results indicate that short-term low-dose dietary supplementation with IMOs does not have adverse effects on the colon tissue of healthy rats.

Diamine oxidase is an intracellular enzyme that catalyses the oxidation of diamines and is a reliable indicator of the integrity of the intestinal mucosa. D-lactic acid is a metabolite of gut microbes and is present in the intestinal lumen. When the intestinal barrier function is damaged and the permeability increases, D-LA will reach the blood, and thus, the content of D-LA in the serum is an indicator of intestinal permeability. The serum DAO and D-LA levels of the four groups of rats are shown in Figures 3E,F. Serum DAO and D-LA levels in DMH group were significantly higher than those in CON group (*p* < 0.05), which confirms that ECC in the rats led to an increase in colonic epithelial permeability and impaired colonic function. However, dietary supplementation with IMOs significantly reduced the increase in serum DAO and D-LA levels in ECC model rats (*p* < 0.05) and mitigated the increase in colonic epithelial permeability in ECC model rats (Figures 3E,F). Compared with the CON group, there were no significant changes in serum DAO and D-LA in healthy rats supplemented with IMOs, indicating that short-term low-dose feeding of IMOs to healthy rats does not have a significant effect on colonic permeability.

PAS staining is a commonly used histochemical staining method; it is generally used to display glycogen and other polysaccharides. Glycogen and goblet cells are purplish red, nuclei are stained blue with haematoxylin, and other tissues are pale pink. In this study, compared with the CON group, the PAS-stained purple-red colour of the colon tissue of the DMH group was reduced, suggesting that the expression of colonic glycogen in the ECC model rats was reduced. Dietary supplementation with IMOs mitigated this phenomenon, as shown in Figure 3G. The expression of colonic glycogen was abundant in the IMOs group, which demonstrates that short-term low-dose dietary supplementation with IMOs has no significant effect on the expression of colonic glycogen in rats.

In addition, this study investigated the effect of dietary supplementation with IMOs on the expression levels of β-catenin, COX-2 and iNOS in the colon tissue of rats with precancerous lesions. The expression of reactive protein in the colon tissue was observed by immunohistochemical staining and image analysis, and the results are shown in Figure 2E. The expression levels of β-catenin and iNOS in the DI group were significantly lower than those in the DMH group (*p* < 0.05). Dietary supplementation with IMOs in the ECC model rats significantly reduced the expression of β-catenin and iNOS in the colon.

### Effects of isomaltooligosaccharides supplementation on the faecal microbiota of rats

After 12 weeks of standard or IMOs-supplemented diets, fresh faeces were collected to extract DNA, and amplification, purification and sequencing of the V3-V4 hypervariable regions of bacterial 16s rRNA was performed on the faeces of rats. The number of clean tags after quality control was between 38,060 and 41,891, and the valid tags were obtained by removing the chimera of clean tags. The number of valid tags was between 28,398 and 36,975, the average length of valid tags was between 421.66 and 429.03 bp, and the number of OTUs in each sample was between 681 and 934 (OTUs were obtained according to a sequence similarity greater than 97%). Annotate species based on representative OTU sequences.

**FIGURE 4:**
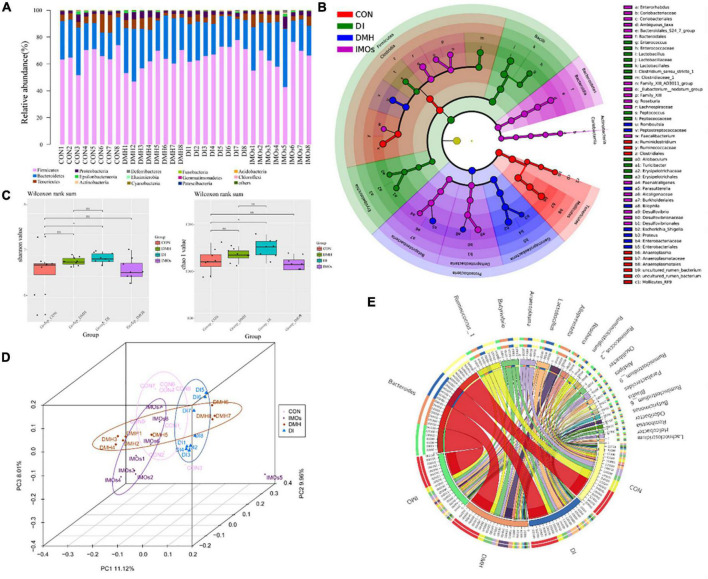
Faecal microbiota effects of DMH treatment after 12 weeks of intervention with or without IMOs diet. **(A)** Relative abundances of phyla in the different groups. **(B)** Taxonomic abundance analysis (LEfSe analysis) revealed multiple differentially abundant taxa in different groups of faeces. **(C)** Microbial richness estimates (Chao index) and diversity indices (Shannon–Wiener) in the different groups. **(D)** PCoA shows separation of the faecal bacterial compositions among the four groups. **(E)** The chord diagram reveals the top 25 abundant genera (abundance > 0.1%) in the four groups.

The bacteria belonged to 15 phyla: Firmicutes, Bacteroidetes, Tenericutes, Proteobacteria, Epsilonbacteraeota, Actinobacteria, Deferribacteres, Elusimicrobia, Cyanobacteria, Fusobacteria, Gemmatimonadetes, Patescibacteria, Acidobacteria, Chloroflexi and others (Figure 4A). Firmicutes was the most abundant among the different genera, and Bacteroides was the most abundant among the four groups of faeces. Linear discriminant analysis effect size (LEfSe) was used to identify the differential abundance of bacterial genera among the four groups. At the family level, Peptostreptococcaceae and Enterobacteriaceae were more abundant in the DMH group, whereas Enterococcaceae, Lactobacillaceae, Clostridiaceae_1, Peptococcaceae and Erysipelotrichaceae were more abundant in the DI group (Figure 4B). At the genus level, Romboutsia, Parasutterella, Bilophila and Proteus were more abundant in the DMH group, while Enterococcus, Lactobacillus, Peptococcus, Allobaculum and Turicibacter were more abundant in the DI group (Figure 4B). The α-diversity of the microbial communities was measured according to the diversity value (Shannon index) and richness estimate (Chao 1 index). The diversity value and the richness estimate of the microbial community in the DI group were significantly higher than those of the CON group (Figure 4C). PCA was used to examine the bacterial diversity among the four groups; 29.09% of the total variance was explained by three principal components (PC1, PC2, and PC3), which were stable and reliable (Figure 4D). A chord diagram was used to depict the top 20 abundant genera in the four groups (Figure 4E). As can be seen, Bacteroides, Ruminococcus_1 and Butyrivibrio were the three predominant taxa in all the groups, accounting for 1.96–14.88% of the total OTUs. The differential abundance among the four groups showed that IMOs can alter the pathological changes in ECC and the structure of the gut microbiome in rats.

### Effects of isomaltooligosaccharides supplementation on faecal metabolite profiles in rats

**FIGURE 5:**
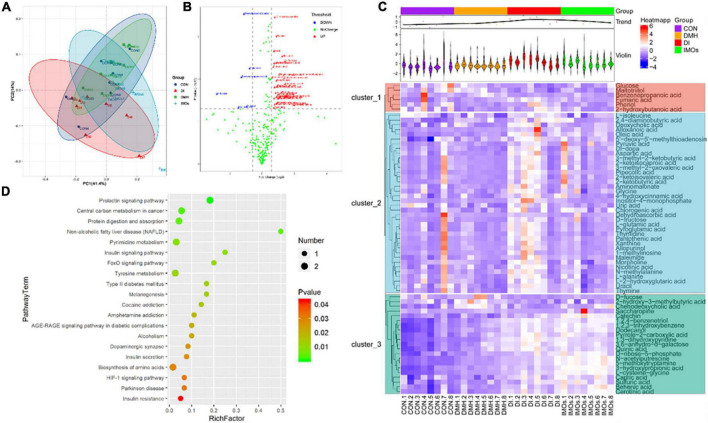
Faecal metabolic profiling of the CON, DMH, DI and IMOs groups. **(A)** Scatter plot of PCoA scores for different groups. **(B)** Volcano plots present differential variables between the DMH and DI groups. Each metabolite is represented by a dot, where the red dots indicate up-regulation, the blue dots indicate down-regulation, and the green dots indicate no statistically significant difference. **(C)** Heatmap of 65 differentially abundant metabolites at normalized levels (*p* < 0.05) among the four groups. The dendrogram shows the distances of the metabolites based on their relative abundances. The normalized abundance values are depicted visually from red to blue, representing the highest and lowest abundances, respectively. **(D)** Enriched KEGG pathways in the DMH group compared with the DI group. The statistical significance values (*p* < 0.05) are depicted visually from red to green, representing the most and least differences, respectively. The dot sizes on the vertical axis represent the metabolite counts in the metabolic pathways.

Faecal metabolic profiling provides a functional readout of the gut microbiota that can be used to understand the underlying mechanisms of the microbiota’s impact on host health (33). A total of 338 peaks were identified and 267 metabolites persisted after background noise was removed using an interquartile range denoising method. Missing values in the original data are replaced with half of the minimum. PCA (Figure 5A) was used to show the distribution of the data showing the separation of faecal metabolite structures between the four groups. Specifically, PC1 and PC2 accounted for 41.40 and 14.00%, of the total variance, respectively. Variables with different metabolites between DMH and DI groups are shown in the form of volcano plots (Figure 5B); each dot represents a metabolite: red dots represent up-regulated metabolites, blue dots represent down-regulated metabolites, and green dots represent non-significantly different metabolites (*p* > 0.05). However, the volcano is complex due to the many variables involved. Therefore, 42 differential metabolites between the DMH and DI groups were identified based on variable importance > 1.0 for projected (VIP) values from orthogonal partial least squares discriminant analysis and *p*-value < 0.05 for Student’s *t*-test. In total, 35 metabolites, including chlorogenic acid, fumaric acid and 1-methylinosine, were significantly reduced abundance in the faeces of DI-treated rats, while the abundance of seven metabolites, including deoxycholic acid (DCA), chenodeoxycholic acid and glucose, were significantly increased compared to the faeces of DMH-treated rats.

The metabolites that differed significantly between the DMH and DI groups were assigned to metabolic pathways based on the Kyoto Encyclopaedia of Genes and Genomes (KEGG) database. This analysis indicated that the differentially abundant metabolites in the DI group were all related to the prolactin signalling pathway, central carbon metabolism in cancer, protein digestion and absorption, non-alcoholic fatty liver disease, and the insulin signalling pathway (*p* < 0.01, frequency-distance relationship correction; Figure 5D).

### Correlation analysis between faecal microbiota and metabolites

**FIGURE 6:**
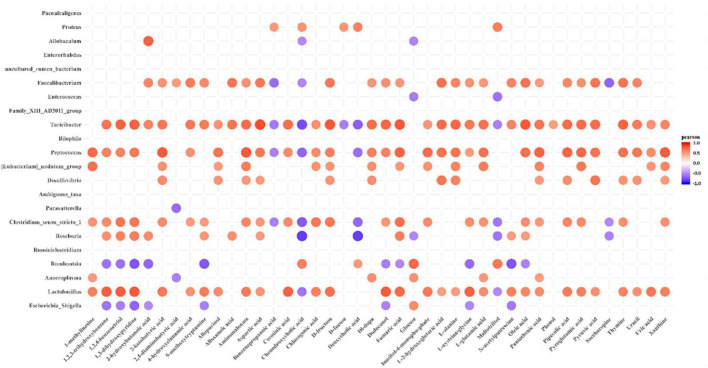
Pearson’s correlation analysis of faecal microbiota and metabolites. *p*-values are plotted from blue to red, where blue represents positive correlation and red represents negative correlation.

Determining whether the composition of the gut microbiota correlates with metabolic profiles, Pearson’s correlation analysis was performed. The metabolic association heatmap (Figure 6) demonstrates that there were Positive and negative correlations between metabolite levels and identified bacterial taxa. The dominant genera in the DMH rats (Figure 4B), such as *Romboutsia* and *Proteus*, displayed strong positive correlations with chenodeoxycholic acid, DCA and maltotriitol. The dominant genera in the DI rats, including *Lactobacillus*, *Peptococcus* and *Turicibacter*, displayed strong positive correlations with 1,2,3-trihydroxybenzene, 1,2,4-benzenetriol, 1,3-dihydroxypyridine, 2-ketobutyric acid, 4-hydroxycinnamic acid, allopurinol, aspartic acid, cerotinic acid, chlorogenic acid, dodecanol, fumaric acid, L-glutamic acid, pipecolic acid, pyroglutamic acid and pyruvic acid. These three genera were also negatively correlated with chenodeoxycholic acid. Overall, dietary IMOs altered bacterial composition and significantly altered the faecal metabolome of DI-treated rats.

## Discussion and conclusion

With the continual agieng of the population, the incidence of colorectal-related diseases is rapidly increasing (34). The prevention, diagnosis and treatment of gastrointestinal diseases such as constipation, inflammatory bowel disease and CC have also increasingly attracted the attention of medical workers and researchers. Studies have shown that specific diets can prevent CC, and diet control and management can reduce the incidence of CC (3). Natural IMOs are usually present in fermented foods. In Asia, IMOs has been used as functional food and sweetener for more than 3 decades (35). IMOs is widely used and safe, and can be used in the production of children’s food (36, 37). They are functional oligosaccharides that cannot be decomposed by human digestive enzymes. They can directly reach the intestines for use by microorganisms and can improve the immune response and regulate intestinal flora. They are a type of prebiotic. Extensive research demonstrates that prebiotics can improve the physiological state of the body by regulating the structure of the intestinal microflora. Animal experiments have shown that IMOs can promote the proliferation of intestinal probiotics and inhibit pathogenic bacteria (38), thereby enhancing intestinal health (39). In addition, IMOs can regulate the immune function of the body. Dietary intake of IMOs can stimulate the production of IL-12 by monocytes, macrophages and dendritic cells, and promote the helper T cell type 1 immune response (40). However, to the best of our knowledge, the use of IMOs to intervene in ECC rats has not yet been reported.

In-depth research on the occurrence and development of CC has demonstrated that ACF are morphological lesions present in the colonic mucosa in ECC. Previous studies have indicated that a high-dose (20%) Galactooligosaccharides (GOS) diet is protective against CC development (41). The current study demonstrated that a relatively low dose (5%) of IMOs has a significant preventive effect on the formation of ACF, a marker of ECC (Figure 3A). It has been reported that reduced ACF in the colons of prebiotic-treated animals may be due to changes in the gut microbial structure, resulting in increased concentrations of SCFAs and decreased pH of the colonic environment, which are important factors in maintaining normal colonic epithelial cell development (42).

The results of the current study showed that dietary supplementation with IMOs had a significant lowering effect on the pH of rat colonic contents (Figure 3B). Soler et al. (43) reported that increased permeability of the colonic epithelium and decreased barrier function are the precursors to colon tumorigenesis. Studies have also shown that feeding IMOs effectively prevents the adverse effects of a high-fat diet on colon structure (38). Thus, in this study, we evaluated the intestinal permeability and barrier function of rats. The results revealed that the levels of DAO and D-LA in the serum of rats in the DMH group were increased, the intestinal permeability was increased, the secretion of glycogen from colonic goblet cells was decreased, and the mechanical barrier of the colonic mucosa was damaged. Dietary supplementation with IMOs (DI group) decreased the serum levels of DAO and D-LA and increased colonic glycogen secretion, significantly improving the barrier function of the rat colon (Figures 3E,F).

Inflammation plays an important role in the development and progression of cancer. Likewise, inflammation, as well as the immune response, are thought to be important factors in CC development (44). Therefore, the levels of inflammatory factors in the serum of ECC rats and the expression levels of inflammatory response-related proteins in colon tissue were investigated. It was confirmed that the serum levels of TNF-α, IL-1β, IL-6 and IL-8 were significantly increased in ECC rats (Figure 2), and the expression levels of the COX-2 and iNOS proteins in colon tissue were significantly increased (Figure 2E). This indicates that the inflammatory factors TNF-α, IL-1β, IL-6, and IL-8 and related enzymes in the inflammatory response, such as COX-2 and iNOS, were involved in the occurrence and development of ECC in rats. Dietary supplementation with IMOs significantly decreased the above indicators. Together, these results showed that IMOs can inhibit the development of ECC by reducing the level of related inflammatory factors and the expression of inflammation-related enzymes.

Previous studies have shown that exogenous enzymes such as β-glucuronidase and β-glucosidase are involved in the transformation of endogenous toxins and genotoxic compounds (30). β-glucuronidase and β-glucosidase are thought to be involved in the occurrence and development of tumours (45). The effects of prebiotics on the activity of exogenous enzymes in the colon have been reported. For example, inulin, lactulose and GOS have been found to significantly reduce the activities of β-glucuronidase and β-glucosidase in animals (46, 47). The current experimental results were similar to those reported previously, whereby dietary supplementation with IMOs significantly reduced the activities of exogenous enzymes (β-glucuronidase and β-glucosidase) in rat faeces (Figures 2F,G). β-glucuronidase and β-glucosidase are produced by Bacteroides, Clostridium and Enterobacteriaceae in the gut (48). The previous study (49) found that the activities of exogenous enzymes produced by probiotics and harmful bacteria in the gut were different. Compared with Bacteroides, Clostridium and Enterobacteriaceae, the activities of exogenous enzymes produced by *Bifidobacterium* and *Lactobacillus* were lower. Therefore, we investigated the effect of dietary supplementation with IMOs on the faecal microbial structure in rats.

The analysis of the rat faecal microbiome showed that the relative abundance of *Lactobacillus* in the intestines of rats in the DI and IMOs groups was significantly higher than that in the CON and DMH groups (Figure 4). This indicates that dietary supplementation with IMOs, a potent *Lactobacillus* factor, could significantly promoted the growth of *Lactobacillus*, which is consistent with previous findings (38). In addition, the current research results are unclear as to whether IMOs can promote the proliferation of *Bifidobacterium*. In this study, there were no significant differences in the relative intestinal abundance of *Bifidobacterium* among the four groups of rats, but there was a downward trend in *Bifidobacterium* in the rats supplemented with IMOs. Studies have shown that feeding IMOs can promote the proliferation of *Bifidobacterium* in the guts of BALB/c mice (50). It has also been reported that dietary supplementation with IMOs reduces *Bifidobacterium* in the guts of rats (51). Such contradictory results may be related to the subjects, doses ingested, and length of the intervention.

Research indicates that the intestinal flora of patients with CC is pathologically imbalanced, and the abundance of *Fusobacterium* in the intestines is increased. In addition, the marker bacteria in the intestines are different in different stages of CC. In ECC, *Fusobacterium* is the main marker bacteria (52). This is consistent with our experimental results, where the relative abundance of *Fusobacterium* in the intestines of rats in the DMH group was significantly higher than that in the other three groups. Dietary supplementation with IMOs can effectively inhibit the proliferation of *Fusobacterium*.

There is growing recognition of the importance of gut microbial metabolites in regulating host metabolism and immune responses (53). In the present experiment, we modulated the gut microbial structure in ECC rats by dietary supplementation with IMOs and alleviated colon-related adverse effects. The role of metabolites cannot be ignored. Intestinal markers differ at different stages of CC, and research suggests that there are increased levels of bile acids and amino acids in the gut in ECC (52). This is consistent with the current findings (Figure 5). The levels of bile acids (including deoxycholic acid and chenodeoxycholic acid) and amino acids (L-glutamic acid and L-alanine) in the intestines of rats with ECC were significantly increased, and dietary supplementation with IMOs significantly alleviated this condition. Moreover, in rats fed IMOs alone, the level of chenodeoxycholic acid was also found to be reduced, indicating that dietary supplementation with IMOs can improve the metabolism of rats.

Dietary supplementation with IMOs increased the succinic acid content of the faeces of healthy rats, and this may be related to the elevated relative abundances of *Roseburia* and *Lactobacillus* in the faeces. Studies have shown that dietary modification can increase the relative abundance of *Roseburia*, a butyrate-producing genus, in faeces (54). Earlier studies also indicated that SCFAs, especially butyric acid, inhibit CC cell growth and are involved in the induction of CC cell apoptosis (55). The present study found that dietary supplementation with IMOs increased faecal butyric acid content and has the potential to prevent ECC.

This work could advance the development of dietary supplements for CRC and could help guide CRC patients to improve their condition through dietary therapy. However, human CRC risk factors are complex. The favourable effect of IMOs on human CRC warrants further clinical studies.

## Data availability statement

The datasets presented in this study can be found in online repositories. The names of the repository/repositories and accession number(s) can be found below: NCBI, PRJNA872320.

## Ethics statement

The animal study was reviewed and approved by Ethics Committee of the Shandong University of Traditional Chinese Medicine. Written informed consent was obtained from the owners for the participation of their animals in this study.

## Author contributions

XC and SL: conceptualization. CL and JC: methodology. BY: software. XL and CW: validation. XC: formal analysis, writing—original draft preparation, supervision, and project administration. ZhenZ: data curation. ZhengZ: writing—review and editing. JY: visualization. XC, JY, and ZhengZ: funding acquisition. All authors have read and agreed to the published version of the manuscript.

## References

[1] Kulshrestha R Tiwari S. Diet and colon cancer: a comprehensive review. In: Vishvakarma NK Nagaraju GP Shukla D editors. *Colon Cancer Diagnosis and Therapy.* (Vol. 2), Cham: Springer International Publishing (2021). p. 53–71.

[2] Rowland I Gibson G Heinken A Scott K Swann J Thiele I Gut microbiota functions: metabolism of nutrients and other food components. *Eur J Nutr.* (2018) 57:1–24. 10.1007/s00394-017-1445-8 28393285 PMC5847071

[3] Yang J Yu J. The association of diet, gut microbiota and colorectal cancer: what we eat may imply what we get. *Protein Cell.* (2018) 9:474–87. 10.1007/s13238-018-0543-6 29713943 PMC5960467

[4] Ganesan K Jayachandran M Xu B. Diet-derived phytochemicals targeting colon cancer stem cells and microbiota in colorectal cancer. *Int J Mol Sci.* (2020) 21:3976. 10.3390/ijms21113976 32492917 PMC7312951

[5] Yamada T. Metagenomic and metabolomic analyses reveal distinct stage-specific phenotypes of the gut microbiota in colorectal cancer. *BIO Web Conf.* (2021) 41:01001. 10.1051/bioconf/20214101001 31171880

[6] Zhang Z Zhao J Tian C Chen X Li H Wei X Targeting the gut microbiota to investigate the mechanism of lactulose in negating the effects of a high-salt diet on hypertension. *Mol NutrFood Res.* (2019) 63:e1800941. 10.1002/mnfr.201800941 30825362

[7] Zhang Z Chen X Cui B. Modulation of the fecal microbiome and metabolome by resistant dextrin ameliorates hepatic steatosis and mitochondrial abnormalities in mice. *Food Funct.* (2021) 12 (Suppl 1):4504–18. 10.1039/D1FO00249J 33885128

[8] Hu Y Winter V. In vitro digestibility of commercial and experimental isomalto-oligosaccharides. *Food Re Int.* (2020) 134:109250. 10.1016/j.foodres.2020.109250 32517953

[9] Hooton D Lentle R Monro J Wickham M Simpson R. The secretion and action of brush border enzymes in the mammalian small intestine. *Rev Physiol Biochem Pharmacol.* (2015) 168:59–118. 10.1007/112_2015_24 26345415

[10] Lee B-H Rose D Lin A Quezada-Calvillo R Nichols B Hamaker B. Contribution of the individual small intestinal α-glucosidases on digestion of unusual α-linked glycemic disaccharides. *J Agric Food Chem.* (2016) 64:6487–94. 10.1021/acs.jafc.6b01816 27480812

[11] Subhan F Hashemi Z Archundia C Turner K Windeler S Gänzle M Ingestion of isomalto-oligosaccharides stimulates insulin and incretin hormone secretion in healthy adults. *J Funct Foods.* (2019) 65:103730. 10.1016/j.jff.2019.103730

[12] Koleva P Ketabi A Valcheva R Dieleman L. Chemically defined diet alters the protective properties of fructo-oligosaccharides and isomalto-oligosaccharides in HLA-B27 transgenic rats. *PLoS One.* (2014) 9:e111717. 10.1371/journal.pone.0111717 25369019 PMC4219767

[13] Wang W Xin H Fang X Dou H Liu F Huang D Isomalto-oligosaccharides ameliorate visceral hyperalgesia with repair damage of ileal epithelial ultrastructure in rats. *PLoS One.* (2017) 12:e0175276. 10.1371/journal.pone.0175276 28437458 PMC5402968

[14] Chen HL Lu Y-H Lin J Jr. Ko L-Y. Effects of isomalto-oligosaccharides on bowel functions and indicators of nutritional status in constipated elderly men. *J Am Coll Nutr.* (2001) 20:44–9. 10.1080/07315724.2001.10719013 11294172

[15] Wang H-F Lim PS Kao M-D Chan E-C Lin L-C Wang N-P. Use of isomalto-oligosaccheride in the treatment of lipid profiles and constipation in hemodialysis patiens. *J Ren Nutr.* (2001) 11:73–9. 10.1016/S1051-2276(01)92591-9 11295027

[16] Gunasekaran S Venkatachalam K Namasivayam N. Anti-inflammatory and anticancer effects of p-methoxycinnamic acid, an active phenylpropanoid, against 1,2-dimethylhydrazine-induced rat colon carcinogenesis. *Mol Cell Biochem.* (2019) 451:117–29. 10.1007/s11010-018-3398-5 29980883

[17] Bartolomeu A Romualdo G Lisón C Besharat ZM Corrales J Chaves M Caffeine and chlorogenic acid combination attenuate early-stage chemically induced colon carcinogenesis in mice: involvement of oncomiR miR-21a-5p. *Int J Mol Sci.* (2022) 23:6292. 10.3390/ijms23116292 35682971 PMC9181067

[18] Kumaraswamy D Kamaleeswari M Murugan S Nalini N. Dose dependent inhibitory effect of dietary caraway on 1,2-dimethylhydrazine induced colonic aberrant crypt foci and bacterial enzyme activity in rats. *Invest New Drugs.* (2006) 24:479–88. 10.1007/s10637-006-6801-0 16598436

[19] McIntosh F Maison N Holtrop G Young P Currie V Ince J Phylogenetic distribution of genes encoding β-glucuronidase activity in human colonic bacteria and the impact of diet on faecal glycosidase activities. *Environ Microbiol.* (2012) 14:1876–87. 10.1111/j.1462-2920.2012.02711.x 22364273

[20] Depenbusch BE Nagaraja TG Sargeant JM Drouillard JS Corrigan ME. Influence of processed grains on fecal pH, starch concentration, and shedding of *Escherichia coli* O157 in feedlot cattle. *J Anim Sci.* (2008) 86:632–9. 18042815 10.2527/jas.2007-0057

[21] Mao JW Huang YS Tang HY Bi J Liu YF Wang YD. Flt3/Flt3L participates in the process of regulating dendritic cells and regulatory T cells in DSS-induced colitis. *Gastroenterol Res Pract.* (2014) 2014:483578. 10.1155/2014/483578 25371672 PMC4209836

[22] Bolger A Lohse M Usadel B. Trimmomatic: a flexible trimmer for illumina sequence data. *Bioinformatics.* (2014) 30:2114–20. 10.1093/bioinformatics/btu170 24695404 PMC4103590

[23] Reyon D Tsai S Khayter C Foden J Sander J Joung J. FLASH assembly of TALENs for high-throughput genome editing. *Nat Biotechnol.* (2012) 30:460–5. 10.1038/nbt.2170 22484455 PMC3558947

[24] Caporaso JG Kuczynski J Stombaugh J Bittinger K Bushman FD Costello EK QIIME allows analysis of high-throughput community sequencing data. *J Nat Methods.* (2010) 7:335–6. 10.1038/nmeth.f.303 PMC3156573 20383131

[25] Edgar RC. UPARSE: highly accurate OTU sequences from microbial amplicon reads. *Nat Methods.* (2013) 10:996–8. 23955772 10.1038/nmeth.2604

[26] Wang Q Garrity GM Tiedje JM Cole JR. Naive Bayesian classifier for rapid assignment of rRNA sequences into the new bacterial taxonomy. *Appl Environ Microbiol.* (2007) 73:5261–7. 10.1128/AEM.00062-07 17586664 PMC1950982

[27] Zhang Z Liu J Li M Yang B Liu W Chu Z *Lactobacillus rhamnosus* encapsulated in alginate/chitosan microgels manipulates the gut microbiome to ameliorate salt-induced hepatorenal injury. *Front Nutr.* (2022) 9:872808. 10.3389/fnut.2022.872808 35495927 PMC9047548

[28] Song Y-B Lamothe L Nava Rodriguez N Rose D Lee B-H. New insights suggest isomaltooligosaccharides are slowly digestible carbohydrates, rather than dietary fibers, at constitutive mammalian α-glucosidase levels. *Food Chem.* (2022) 383:132456. 10.1016/j.foodchem.2022.132456 35182873

[29] Lin WW Karin M. A cytokine-mediated link between innate immunity, inflammation, and cancer. *J Clin Invest.* (2007) 117:1175–83. 17476347 10.1172/JCI31537 PMC1857251

[30] Macfarlane GT Macfarlane S. Bacteria, colonic fermentation, and gastrointestinal health. *J AOAC Int.* (2012) 95:50–60. 10.5740/jaoacint.sge_macfarlane 22468341

[31] Keawyok K. Nutritionally-complete formula fortified with isomalto-oligosaccharide for hemodialysis patients. *Funct Foods Health Dis.* (2020) 10:290–304. 10.31989/ffhd.v10i7.716

[32] Kohno H Suzuki R Sugie S Tanaka T. Beta-catenin mutations in a mouse model of inflammation-related colon carcinogenesis induced by 1,2-dimethylhydrazine and dextran sodium sulfate. *Cancer Sci.* (2010) 96:69–76. 10.1111/j.1349-7006.2005.00020.x 15723650 PMC11159258

[33] Jonas Z Jackson MA Gabi K Massimo M Long T Amalio T The fecal metabolome as a functional readout of the gut microbiome. *Nat Genet.* (2018) 50:790–5. 29808030 10.1038/s41588-018-0135-7 PMC6104805

[34] Siegel R Miller K Goding Sauer A Fedewa S Butterly L Anderson J Colorectal cancer statistics, 2020. *CA Cancer J Clin.* (2020) 70:145–64. 10.3322/caac.21601 32133645

[35] Goffin D Delzenne N Blecker C Hanon E Deroanne C Paquot M. Will isomalto-oligosaccharides, a well-established functional food in Asia, break through the European and American market? The status of knowledge on these prebiotics. *Crit Rev Food Sci Nutr.* (2011) 51:394–409. 10.1080/10408391003628955 21491266

[36] Jiang X. Application of isomalto-oligosaccharides used in children with functional dyspepsia. *J Med Forum.* (2005).

[37] Sorndech W Nakorn K Tongta S Blennow A. Isomalto-oligosaccharides: recent insights in production technology and their use for food and medical applications. *LWT.* (2018) 95:135–42. 10.1016/j.lwt.2018.04.098

[38] Singh D Singh J Boparai R Zhu J Mantri S Khare P Isomalto-oligosaccharides, a prebiotic, functionally augment green tea effects against high fat diet-induced metabolic alterations via preventing gut dysbacteriosis in mice. *Pharmacol Res.* (2017) 123:103–13. 10.1016/j.phrs.2017.06.015 28668709

[39] Li T Lu X Yang X. Evaluation of clinical safety and beneficial effects of stachyose-enriched α-galacto-oligosaccharides on gut microbiota and bowel function in human. *Food Funct.* (2016) 8:262–9. 10.1039/C6FO01290F 28001151

[40] Mizubuchi H Yajima T Aoi N Tomita T Yoshikai Y. Isomalto-oligosaccharides polarize Th1-like responses in intestinal and systemic immunity in mice. *J Nutr.* (2005) 135:2857–61. 10.1093/jn/135.12.2857 16317132

[41] Wijnands M Schoterman HC Bruijntjes J Hollanders VMH Woutersen R. Effect of dietary galacto-oligosaccharides on azoxymethane-induced aberrant crypt foci and colorectal cancer in Fischer 344 rats. *Carcinogenesis.* (2001) 22:127–32. 10.1093/carcin/22.1.127 11159750

[42] Aachary A Gobinath D Srinivasan K Siddalingaiya P. Protective effect of xylooligosaccharides from corncob on 1,2-dimethylhydrazine induced colon cancer in rats. *Bioact Carbohydr Diet Fibre.* (2015) 5:146–52. 10.1016/j.bcdf.2015.03.004

[43] Soler AP Miller R Laughlin K Carp N Klurfeld D Mullin J. Increased tight junctional permeability is associated with the development of colon cancer. *Carcinogenesis.* (1999) 20:1425–31. 10426787 10.1093/carcin/20.8.1425

[44] Chang P-H Pan Y-P Fan C-W Tseng W-K Huang J-S Wu T Pretreatment serum interleukin-1β, interleukin-6, and tumor necrosis factor-α levels predict the progression of colorectal cancer. *Cancer Med.* (2016) 5:426–33. 10.1002/cam4.602 26799163 PMC4799955

[45] Goldin B Gorbach SL. The relationship between diet and rat fecal bacterial enzymes implicated in colon cancer. *J Natl Cancer Inst.* (1976) 57:371–5. 10.1093/jnci/57.2.371 1003518

[46] Qamar T Syed F Nasir M Rehman H Zahid M Liu R Novel combination of prebiotics galacto-oligosaccharides and inulin-inhibited aberrant crypt foci formation and biomarkers of colon cancer in wistar rats. *Nutrients.* (2016) 8:465. 10.3390/nu8080465 27490566 PMC4997378

[47] Verma A Shukla G. Administration of prebiotic inulin suppresses 1,2 dimethylhydrazine dihydrochloride induced procarcinogenic biomarkers fecal enzymes and preneoplastic lesions in early colon carcinogenesis in sprague dawley rats. *J Funct Foods.* (2013) 5:991–6. 10.1016/j.jff.2013.02.006

[48] Raman M Ambalam P Kondepudi KK Pithva S Kothari C Patel A Potential of probiotics, prebiotics and synbiotics for management of colorectal cancer. *Gut Microbes.* (2013) 4:181–92. 10.4161/gmic.23919 23511582 PMC3669163

[49] Mital B Garg S. Anticarcinogenic, hypocholesterolemic, and antagonistic activities of lactobacillus acidophilus. *Crit Rev Microbiol.* (1995) 21:175–214. 10.3109/10408419509113540 8845062

[50] Kaneko T Kohmoto T Fukui F Akiba T Suzuki S Hirao A Acute and chronic toxicity and mutagenicity studies on Isomaltooligosaccharides, and the effects on peripheral blood lymphocytes and intestinal microflora. *Food Hygiene Safe Sci.* (1990) 31:394–403. 10.3358/shokueishi.31.394 15018534

[51] Ketabi A Dieleman L. Influence of isomalto-oligosaccharides on intestinal microbiota in rats. *J Appl Microbiol.* (2011) 110:1297–306. 10.1111/j.1365-2672.2011.04984.x 21338450

[52] Yachida S Mizutani S Hirotsugu S Shiba S Nakajima T Sakamoto T Metagenomic and metabolomic analyses reveal distinct stage-specific phenotypes of the gut microbiota in colorectal cancer. *Nat Med.* (2019) 25:1–9. 10.1038/s41591-019-0458-7 31171880

[53] Schröder B. The gut microbiota and host metabolism. In: Rook GAW Lowry CA editors. *Evolution, Biodiversity and a Reassessment of the Hygiene Hypothesis. Progress in Inflammation Research.* (Cham: Springer) (2022). p. 141–75.

[54] Kasahara K Krautkramer K Org E Romano K Kerby R Vivas E Interactions between Roseburia intestinalis and diet modulate atherogenesis in a murine model. *Nat Microbiol.* (2018) 3:1461–71. 10.1038/s41564-018-0272-x 30397344 PMC6280189

[55] Fernández J Ledesma E Monte J Millán E Costa P Fuente V Traditional processed meat products re-designed towards inulin-rich functional foods reduce polyps in two colorectal cancer animal models. *Sci Rep.* (2019) 9:14783. 10.1038/s41598-019-51437-w 31616028 PMC6794276

