# Supplementary material for: Isomaltooligosaccharides inhibit early colorectal carcinogenesis in a 1,2-dimethylhydrazine-induced rat model
Source: Front Nutr. 2022 Sep 15;9:995126. doi: 10.3389/fnut.2022.995126 (PMC9521046; doi:10.3389/fnut.2022.995126)
Supplement: Supplementary file 1 [file Data_Sheet_1.docx]

**Supporting information**

**Figure S1. Change in rats body weight during experimental phase.**

**
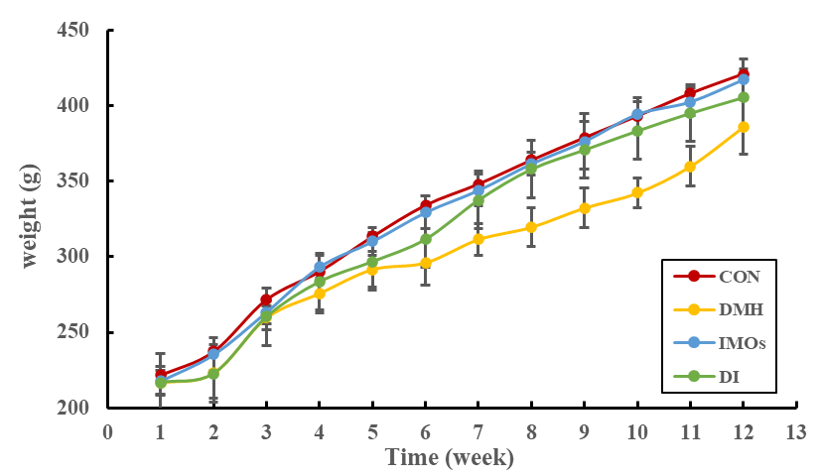
**

**Figure S2. Relative abundances of fifteen phyla in the four groups.**

**
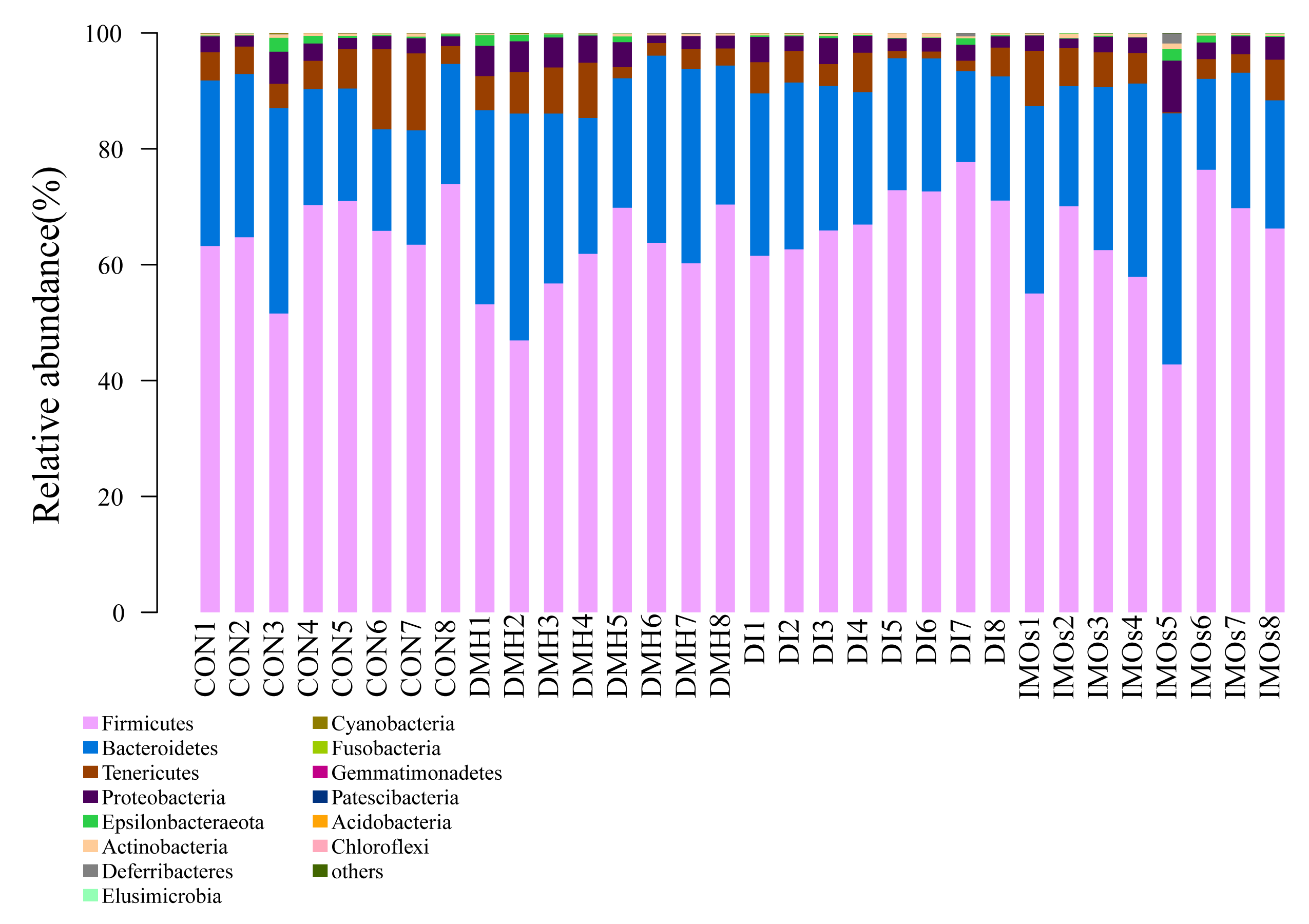
**

**Table S1. Average food and energy intake in four groups of rats throughout the experiment.**

| Group | Food intake（g/Rat/Day） | Energy intake（kJ/Rat/Day） |
| --- | --- | --- |
| CON | 21.2 ± 0.17 | 341.1 ± 2.71 |
| DMH | 21.2 ± 0.29 | 341.9 ± 4.69 |
| DI | 21.4 ± 0.21 | 341.2 ± 3.37 |
| IMOs | 21.5 ± 0.16 | 341.6 ± 2.58 |
